# Mammography screening is associated with more favourable breast cancer tumour characteristics and better overall survival: case-only analysis of 3739 Asian breast cancer patients

**DOI:** 10.1186/s12916-022-02440-y

**Published:** 2022-08-04

**Authors:** Zi Lin Lim, Peh Joo Ho, Alexis Jiaying Khng, Yen Shing Yeoh, Amanda Tse Woon Ong, Benita Kiat Tee Tan, Ern Yu Tan, Su-Ming Tan, Geok Hoon Lim, Jung Ah Lee, Veronique Kiak-Mien Tan, Jesse Hu, Jingmei Li, Mikael Hartman

**Affiliations:** 1 Genome Institute of Singapore, Laboratory of Women’s Health & Genetics, 60 Biopolis Street, Genome, #02-01, Singapore, 138672 Singapore; 2 Saw Swee Hock School of Public Health, National University of Singapore, Singapore, 117549 Singapore; 3 Department of Surgery, Yong Loo Lin School of Medicine, National University of Singapore, Singapore, 117597 Singapore; 4 Department of Surgery, National University Hospital, Singapore, 119054 Singapore; 5 Department of General Surgery, Sengkang General Hospital, Singapore, 544886 Singapore; 6 Department of Breast Surgery, Singapore General Hospital, Singapore, 168753 Singapore; 7 Division of Surgery and Surgical Oncology, National Cancer Centre Singapore, Singapore, 169610 Singapore; 8 Department of General Surgery, Tan Tock Seng Hospital, Singapore, 308433 Singapore; 9 Lee Kong Chian School of Medicine, Nanyang Technology University, Singapore, 308232 Singapore; 10 Division of Breast Surgery, Changi General Hospital, Singapore, 529889 Singapore; 11 Breast Department, KK Women’s and Children’s Hospital, Singapore, 229899 Singapore; 12 Duke-NUS Medical School, Singapore, 169857 Singapore; 13 Department of General Surgery, Ng Teng Fong General Hospital, Singapore, 609606 Singapore

**Keywords:** Breast cancer, Mammography screening, Cancer survival, Sociodemographics and health outcomes

## Abstract

**Background:**

Early detection of breast cancer (BC) through mammography screening (MAM) is known to reduce mortality. We examined the differential effect that mammography has on BC characteristics and overall survival and the sociodemographic determinants of MAM utilization in a multi-ethnic Asian population.

**Methods:**

This study included 3739 BC patients from the Singapore Breast Cancer Cohort (2010–2018). Self-reported sociodemographic characteristics were collected using a structured questionnaire. Clinical data were obtained through medical records. Patients were classified as screeners (last screening mammogram ≤ 2 years before diagnosis), non-screeners (aware but did not attend or last screen > 2years), and those unaware of MAM. Associations between MAM behaviour (MB) and sociodemographic factors and MB and tumour characteristics were examined using multinomial regression. Ten-year overall survival was modelled using Cox regression.

**Results:**

Patients unaware of screening were more likely diagnosed with late stage (OR_stage III vs stage I (Ref)_ [95% CI]: 4.94 [3.45–7.07], *p* < 0.001), high grade (OR_poorly vs well-differentiated (reference)_: 1.53 [1.06–2.20], *p* = 0.022), nodal-positive, large size (OR_>5cm vs ≤2cm (reference)_: 5.06 [3.10–8.25], *p* < 0.001), and HER2-positive tumours (OR_HER2-negative vs HER2-positive (reference)_: 0.72 [0.53–0.97], *p* = 0.028). Similar trends were observed between screeners and non-screeners with smaller effect sizes. Overall survival was significantly shorter than screeners in the both groups (HR_non-screeners_: 1.89 [1.22–2.94], *p* = 0.005; HR_unaware_: 2.90 [1.69–4.98], *p* < 0.001).

Non-screeners and those unaware were less health conscious, older, of Malay ethnicity, less highly educated, of lower socioeconomic status, more frequently ever smokers, and less physically active. Among screeners, there were more reported personal histories of benign breast surgeries or gynaecological conditions and positive family history of breast cancer.

**Conclusions:**

Mammography attendance is associated with more favourable BC characteristics and overall survival. Disparities in the utility of MAM services suggest that different strategies may be needed to improve MAM uptake.

**Supplementary Information:**

The online version contains supplementary material available at 10.1186/s12916-022-02440-y.

## Background

Female breast cancer overtook lung cancer to be the most commonly diagnosed cancer type in the world in 2020, with 2.3 million cases diagnosed worldwide [1]. In the same year, 685,000 breast cancer-related deaths were recorded globally. Early detection of breast cancer when the tumour is small and manageable with less radical treatment is possible with mammography, even before symptoms appear. Mammography screening is currently the most reliable breast cancer screening tool, offering high sensitivity (77 to 95%) and specificity (94 to 97%) in detecting breast abnormalities [2]. Other forms of breast cancer screening exams include ultrasound and MRI. However, mammography is the only approach that has been proven to reduce deaths by breast cancer by early detection in the population-based screening setting [3].

The number of lives saved by mammography screening is substantial. Mammography screening programs in Europe have shown a 25–30% breast cancer mortality reduction in women between 50 and 74 years [4]. In a prospective study of 7301 patients diagnosed with invasive breast cancer by Webb et al., it was found that seven in ten deaths from breast cancer occur in women who have never gone for mammography screening prior to diagnosis (65%) or those not regularly screened according to recommended intervals (6%) [5]. In another large study by Duffy et al. comprising over half a million women residing in Sweden, mammography screening was found to reduce rates of advanced and deadly breast cancers [5]. Women who screened were found to be 41% less likely to die from breast cancer within 10 years, compared to those who did not screen. A 25% reduction in the rate of advanced breast cancers was also observed among screeners compared to non-screeners. The impact of organized mammography screening in the reduction of fatal breast cancers is independent of advances in breast cancer treatment regimens [6].

When a participation rate of 70% within the target population receives mammography, a significant reduction in breast cancer mortality at the population level can be expected after 7–10 years [7]. According to the European guidelines, 70–75% of eligible women should attend the screening. Women of non-European ancestries are known to have lower mammography screening uptake return rates compared to Caucasians [8]. Despite the presence of highly subsidized nationwide mammography screening programmes established in the early 2000s in high-income Asian countries such as Korea, Japan, Taiwan, and Singapore, uptake of screening mammography remains low. The participation rate in Korea was the highest among the three countries with organized mammography screening at 59.7% in 2015 [9]. In 2016, only 44.9% of the target women in Japan had undergone mammography screening within the past 2 years [10]. In Taiwan, the biennial participation rate was slightly below 40% in 2014 [11]. In a similar period (2015–2016), less than 40% of the target population in Singapore attended timely mammography screening according to prevailing guidelines [12]. The low screening uptake and even lower adherence to regular screening is a major public health issue in Singapore [13].

In this large case-only analysis comprising 3739 breast cancer patients in Singapore, we examined the differential effect that mammography screening has on breast cancer characteristics and overall survival, the level of awareness of women on the national screening mammography programme, and the sociodemographic determinants of mammography screening utilization.

## Methods

### Study population

The Singapore Breast Cancer Cohort (SGBCC) is a multicentre cohort study of breast cancer patients in Singapore. Established in 2010, it aims to investigate the associations between various genetic and non-genetic factors and breast cancer risk (cohort profile described in [14]). Patients are recruited across seven public hospitals, namely, National University Hospital (NUH), KK Women’s and Children’s Hospital (KKH), Tan Tock Seng Hospital (TTSH), National Cancer Centre Singapore (NCCS), Singapore General Hospital (SGH), Changi General Hospital (CGH), and Ng Teng Fong General Hospital (NTFGH). The recruiting hospitals collectively treat ~76% of the breast cancer patients in Singapore [14].

Eligible patients have to be (1) diagnosed with breast carcinoma in situ or invasive breast cancer, (2) citizens or permanent residents of Singapore, and (3) aged 21 years and above. As part of the recruitment process, patients completed a structured questionnaire which included questions relating to breast cancer risk factors (i.e. mammography awareness and attendance, reproductive factors and family history of breast cancer, etc.), with assistance as required from a trained study coordinator.

SGBCC was approved by the National Healthcare Group Domain Specific Review Board (reference number: 2009/00501) and the SingHealth Centralised Institutional Review Board (CIRB Ref: 2019/2246 [2010/632/B]). Informed consent was obtained from all patients.

### Mammography behaviour

Information on mammography behaviour was obtained from the questionnaire administered at recruitment. Questions included “Have you heard of mammography before your diagnosis of breast cancer?” and “Have you ever had a mammography exam before your diagnosis of breast cancer? If yes, what year?” Patients were categorized by mammography behaviour based on their answers into unaware (have not heard of mammography before), non-screeners (true non-screeners: have not attended mammography; non-regular screeners: attended mammography but could not recall the year of the last visit/ attended mammography but the last visit was more than 2 years prior to diagnosis), and screeners (attended mammography within 2 years prior to diagnosis). During the administration of the questionnaire, participants were also asked for specific reasons as to why they attended or did not attend mammography. The answers given were captured by the study coordinator and checked off in a list of options given. The list of options was then further categorized based on the primary themes they represent (Additional file 1: Fig. S1).

### Sociodemographic and breast cancer risk factor data

Baseline information on lifestyle and breast cancer risk factors was obtained at the time of recruitment via the structured questionnaire. The variables included ethnicity, physical activity levels, smoking (yes, no, or missing) and alcohol consumption (yes, no, or missing), previous benign lump or gynaecological surgery (yes, no, or missing), family history of breast and ovarian cancer (yes, no, or missing), reproductive factors, etc. Details on how physical activity levels and menopausal status was coded may be found in Additional file 1: Figs. S2 and S3 respectively. Medical history, specifically, previous diagnoses of heart attack, asthma, renal disease, stroke, diabetes, and previous cancer, was also collected. Comorbidities were combined and scored according to the Charlson comorbidity index [15].

Sociodemographic factors were derived from the questionnaire administered at recruitment, where individual factors were further categorized for ease of analysis (Additional file 1: Fig. S4). Housing (HDB 1–3 room flat, HDB >3room flat (4, 5, or executive type), or private) [16], highest qualification achieved (no formal/primary, secondary, post-secondary (non-tertiary), professional diploma, or tertiary), and marital status (married, never married, widowed, or separated/divorced) were used as proxies for economic, education, and social support status, respectively.

### Clinical data

Clinical data on tumour characteristics and treatment modalities were obtained through medical records. The variables included disease stage (stage I, II, III), nodal involvement (yes/no), tumour size (≤2 cm, >2–5 cm, and >5 cm, other/missing), histological grade (well-, moderately, poorly differentiated), oestrogen receptor (ER) status (positive/negative), progesterone receptor (PR) status (positive/negative), human epidermal growth factor receptor 2 (HER2) status (positive/negative), surgery (yes/no), any chemotherapy (neoadjuvant or adjuvant, yes/no), endocrine therapy (yes/no), and radiotherapy (yes/no). Intrinsic-like subtypes were defined using immunohistochemical markers for ER, PR, and HER2 in conjunction with histologic grade: luminal A [ER+/PR+, HER2−, well- or moderately differentiated], luminal B [HER2−] (ER+/PR+, HER2−, and poorly differentiated), luminal B [HER2+] (ER+/PR+, HER2+, and poorly differentiated), HER2-overexpressed [HER2+], and triple-negative [ER−, PR−, and HER2−] [17].

### Passive follow-up

Information on vital status and cause of death was obtained via linkage with the Registry of Births and Deaths using each individual’s unique National Registration Identity Card (NRIC) number [14]. The completeness of the registry is estimated to be over 99% [18]. Hospitals have differing schedules in updating their in-house breast cancer registry, with a collection of variables ending at different years (NUH: 30 April 2017; KKH: 30 June 2017; CGH: 16 April 2018; TTSH: 30 April 2018). For SGH, NCCS, and NTFGH, not all NRICs were sent to the registry at the same time, and the date of follow-up was obtained from the electronic medical records; all recorded deaths are verified with the Registry of Births and Deaths.

### Exclusions

Additional file 1: Fig. S5 summarizes the exclusions performed for this study. We excluded 66 patients without a valid diagnosis data, 9 male patients, 478 patients who were diagnosed before 2002, 3350 patients diagnosed at below 50 years old (i.e. below the target group), 1109 patients without mammography data, 11 patients with invalid mammography date, 272 patients with a missing stage at diagnosis, 771 patients diagnosed at stage 0, 210 patients diagnosed with stage IV cancer, 130 patients without date of the last follow-up, 67 patients without known vital status, and 203 patients with time to study entry more than or equal to 10 years after diagnosis. The analytical cohort comprised 3739 breast cancer patients.

### Statistical analysis

Characteristics of the study population were described by frequency and percentage for categorical variables and by the mean and standard deviation (SD) for continuous variables. The associations between mammography behaviour and patient characteristics were studied using the chi-square test and Kruskal-Wallis test, for categorical and continuous variables, respectively.

The associations between mammography behaviour (screeners, non-screeners, unaware) and disease characteristics were assessed using multinomial logistic regression models (*multinom* function in R package “nnet”), adjusting for age at diagnosis, site, ethnicity, and case type (incident/prevalent). We ran a sensitivity analysis including only incident cases and another separate sensitivity analysis including patients diagnosed with stage 0 or stage IV cancer. The Kaplan-Meier (KM) method was used to analyse all-cause mortality (R package “survival”); survival curves were compared using the log-rank test. In addition, overall survival was studied using Cox proportional hazard models (survival package in R, where the *Surv (time at entry, follow-up time, event)* command was used to estimate hazard ratios (HR) and corresponding 95% confidence intervals (CI)). Time at entry was defined as the time between the date of recruitment and the date of diagnosis. Follow-up time was defined as the time between the date of death/last follow-up date and the diagnosis date, truncated at 10 years post-diagnosis. In the multivariate Cox regression model, the effect of mammography behaviour on survival was adjusted for all factors significantly associated with 10-year overall survival in univariate Cox regression models. Proportional hazards assumptions for the Cox regression model fits were tested using the cox.zph function. Sensitivity analyses were conducted separately for (i) incident-only patients, (ii) study population including stage 0 and stage IV cancer, and (iii) 5-year survival. Further comparisons of disease severity and overall survival between non-regular screeners and true non-screeners were done using multinomial regression and Cox regression respectively.

To assess associations between sociodemographic factors and mammography behaviour (screeners, non-screeners, unaware), multinomial logistic regression models were used, adjusting for age at diagnosis, site, and case type (incident/prevalent). Sensitivity analyses were done for incident-only cases.

We further studied the deterrents and motivators of attending mammography for non-screeners and screeners, respectively. The *Heatmap* function in the R package “ComplexHeatmap” was used to cluster reasons given for attending or not attending mammography screening and to visualize the results with dendrograms. Finally, we examined the sociodemographic factors associated with cues to action (one of the motivators) for mammography screening, using multinomial regression, adjusting for all factors found to be significant in the univariate models.

## Results

### Population description

Table 1 shows the descriptive statistics of patients’ characteristics. Among the 3739 patients included, 1089 (29.1%) were screeners, 2260 (60.4%) were non-screeners, and 390 (10.4%) were unaware of mammography prior to diagnosis. The majority of the patients had secondary school qualification (44.2%), resided in 4-room/5-room/executive type HDB (HDB >3 rooms) (61.7%), and were married (70.9%). Other treatment characteristics that were explored can be found in Additional file 1: Table S1.

**Table 1:** Characteristics of the study population

***n*** (%)	Total, ***n*** = 3739	Screeners, ***n*** = 1089 (29.1)	Non-screeners, ***n*** = 2260 (60.4)	Unaware, ***n*** = 390 (10.4)	***P***
**Site,** ***n*** **(%)**					
CGH	377 (10.1)	79 (7.3)	239 (10.6)	59 (15.1)	<0.001
KKH	724 (19.4)	173 (15.9)	476 (21.1)	75 (19.2)	
NCC	704 (18.8)	260 (23.9)	391 (17.3)	53 (13.6)	
NTFGH	32 (0.9)	7 (0.6)	23 (1.0)	2 (0.5)	
NUH	630 (16.8)	164 (15.1)	398 (17.6)	68 (17.4)	
SGH	318 (8.5)	107 (9.8)	185 (8.2)	26 (6.7)	
TTSH	954 (25.5)	299 (27.5)	548 (24.2)	107 (27.4)	
**Sociodemographic factors**					
**Age at diagnosis (years, IQR)**					
	60.0 (55.0–66.0)	58.0 (54.0–64.0)	60.0 (55.0–66.0)	66.0 (59.0–73.0)	<0.001
**Age at diagnosis (categorical),** ***n*** **(%)**					
50–59	1800 (48.1)	610 (56.0)	1084 (48.0)	106 (27.2)	<0.001
≥60	1939 (51.9)	479 (44.0)	1176 (52.0)	284 (72.8)	
**Ethnicity,** ***n*** **(%)**					
Chinese	3000 (80.2)	888 (81.5)	1797 (79.5)	315 (80.8)	0.016
Malay	431 (11.5)	99 (9.1)	281 (12.4)	51 (13.1)	
Indian	215 (5.8)	72 (6.6)	122 (5.4)	21 (5.4)	
Others	93 (2.5)	30 (2.8)	60 (2.7)	3 (0.8)	
**Highest qualification attained,** ***n*** **(%)**					
No formal/primary	1358 (36.3)	241 (22.1)	837 (37.0)	280 (71.8)	<0.001
Secondary	1651 (44.2)	545 (50.0)	1013 (44.8)	93 (23.8)	
Post-secondary (non-tertiary)	202 (5.4)	84 (7.7)	114 (5.0)	4 (1.0)	
Professional diploma	183 (4.9)	93 (8.5)	88 (3.9)	2 (0.5)	
Tertiary	227 (6.1)	94 (8.6)	131 (5.8)	2 (0.5)	
Missing	118 (3.2)	32 (2.9)	77 (3.4)	9 (2.3)	
**Housing,** ***n*** **(%)**					
HDB 1–3 rooms	912 (24.4)	192 (17.6)	566 (25.0)	154 (39.5)	<0.001
HDB >3 rooms	2308 (61.7)	694 (63.7)	1408 (62.3)	206 (52.8)	
Private	462 (12.4)	193 (17.7)	247 (10.9)	22 (5.6)	
Other/missing	57 (1.5)	10 (0.9)	39 (1.7)	8 (2.1)	
**Marital status,** ***n*** **(%)**					
Married	2651 (70.9)	828 (76.0)	1595 (70.6)	228 (58.5)	<0.001
Never married	469 (12.5)	126 (11.6)	299 (13.2)	44 (11.3)	
Widowed	421 (11.3)	84 (7.7)	244 (10.8)	93 (23.8)	
Separated or divorced	198 (5.3)	51 (4.7)	122 (5.4)	25 (6.4)	
**Lifestyle risk factors**					
**Smoking,** ***n*** **(%)**					
No	3611 (96.6)	1063 (97.6)	2179 (96.4)	369 (94.6)	0.016
Yes	128 (3.4)	26 (2.4)	81 (3.6)	21 (5.4)	
**Alcohol,** ***n*** **(%)**					
No	3633 (97.2)	1055 (96.9)	2194 (97.1)	384 (98.5)	0.251
Yes	106 (2.8)	34 (3.1)	66 (2.9)	6 (1.5)	
**Physical activity between ages 18 and 30 years,** ***n*** **(%)**					
1	264 (7.1)	70 (6.4)	158 (7.0)	36 (9.2)	<0.001
2	2517 (67.3)	673 (61.8)	1541 (68.2)	303 (77.7)	
3	210 (5.6)	78 (7.2)	126 (5.6)	6 (1.5)	
4	455 (12.2)	146 (13.4)	279 (12.3)	30 (7.7)	
5	293 (7.8)	122 (11.2)	156 (6.9)	15 (3.8)	
**Medical risk factors**					
**Charlson comorbidity index,** ***n*** **(%)**					
0	2549 (68.2)	780 (71.6)	1552 (68.7)	217 (55.6)	<0.001
1	852 (22.8)	216 (19.8)	520 (23.0)	116 (29.7)	
>1	334 (8.9)	93 (8.5)	184 (8.1)	57 (14.6)	
Missing	4 (0.1)	0 (0.0)	4 (0.2)	0 (0.0)	
**Previous surgery for benign lump,** ***n*** **(%)**					
No	3301 (88.3)	896 (82.3)	2038 (90.2)	367 (94.1)	<0.001
Yes	435 (11.6)	193 (17.7)	221 (9.8)	21 (5.4)	
Missing	3 (0.1)	0 (0.0)	1 (0.0)	2 (0.5)	
**Previous gynaecological surgery,** ***n*** **(%)**					
No	2302 (61.6)	632 (58.0)	1414 (62.6)	256 (65.6)	0.007
Yes	1431 (38.3)	456 (41.9)	844 (37.3)	131 (33.6)	
Missing	6 (0.2)	1 (0.1)	2 (0.1)	3 (0.8)	
**Family history of breast cancer,** ***n*** **(%)**					
No	2759 (73.8)	760 (69.8)	1687 (74.6)	312 (80.0)	<0.001
Yes	837 (22.4)	294 (27.0)	482 (21.3)	61 (15.6)	
Missing	143 (3.8)	35 (3.2)	91 (4.0)	17 (4.4)	
**Family history of ovarian cancer,** ***n*** **(%)**					
No	3477 (93.0)	1017 (93.4)	2101 (93.0)	359 (92.1)	0.797
Yes	107 (2.9)	32 (2.9)	62 (2.7)	13 (3.3)	
Missing	155 (4.1)	40 (3.7)	97 (4.3)	18 (4.6)	
**Reproductive risk factors**					
**Age at first full-term pregnancy,** ***n*** **(%)**					
Nulliparous	725 (19.4)	200 (18.4)	462 (20.4)	63 (16.2)	<0.001
<20	217 (5.8)	45 (4.1)	118 (5.2)	54 (13.8)	
20–24	808 (21.6)	209 (19.2)	497 (22.0)	102 (26.2)	
25–29	1113 (29.8)	349 (32.0)	661 (29.2)	103 (26.4)	
>30	853 (22.8)	283 (26.0)	510 (22.6)	60 (15.4)	
Missing	23 (0.6)	3 (0.3)	12 (0.5)	8 (2.1)	
**Parity,** ***n*** **(%)**					
0	725 (19.4)	200 (18.4)	462 (20.4)	63 (16.2)	<0.001
1	459 (12.3)	126 (11.6)	295 (13.1)	38 (9.7)	
2	1296 (34.7)	442 (40.6)	762 (33.7)	92 (23.6)	
≥3	1258 (33.6)	321 (29.5)	741 (32.8)	196 (50.3)	
Missing	1 (0.0)	0 (0.0)	0 (0.0)	1 (0.3)	
**Infertility treatment,** ***n*** **(%)**					
No	3590 (96.0)	1028 (94.4)	2178 (96.4)	384 (98.5)	<0.001
Yes	149 (4.0)	61 (5.6)	82 (3.6)	6 (1.5)	
**Oral contraception,** ***n*** **(%)**					
No	2813 (75.2)	818 (75.1)	1698 (75.1)	297 (76.2)	0.906
Yes	926 (24.8)	271 (24.9)	562 (24.9)	93 (23.8)	
**Hormone replacement treatment,** ***n*** **(%)**					
Never	3309 (88.5)	918 (84.3)	2018 (89.3)	373 (95.6)	<0.001
Ever	401 (10.7)	161 (14.8)	227 (10.0)	13 (3.3)	
Missing	29 (0.8)	10 (0.9)	15 (0.7)	4 (1.0)	
**Menopausal status at diagnosis,** ***n*** **(%)**					
Post-menopausal	3084 (82.5)	845 (77.6)	1885 (83.4)	354 (90.8)	<0.001
Pre-menopausal	655 (17.5)	244 (22.4)	375 (16.6)	36 (9.2)	

The *p*-value (*P*) for categorical variables is based on the chi-square test and the *p*-value for continuous variables is based on the Kruskal-Wallis test

*CGH* Changi General Hospital, *KKH* KK Women’s and Children’s Hospital, *NCC* National Cancer Centre, *NTFGH* Ng Teng Fong General Hospital, *NUH* National University Hospital, *SGH* Singapore General Hospital, *TTSH* Tan Tock Seng Hospital, *IQR* interquartile range

**Fig. 1:**
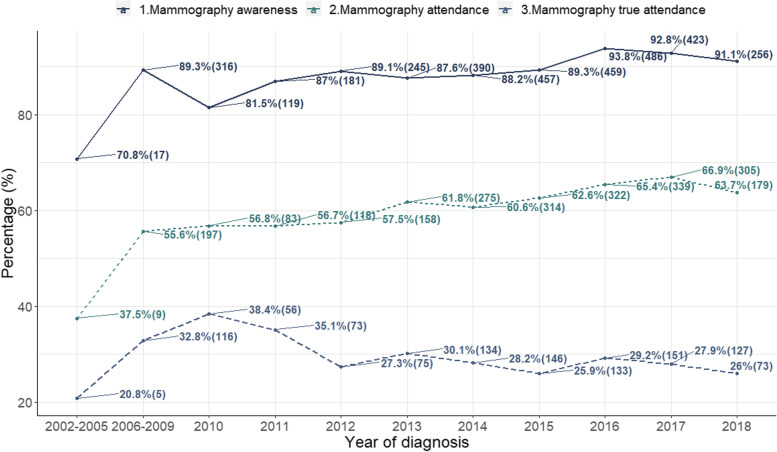
Mammography awareness and attendance among eligible participants diagnosed from 2002 to 2018. Despite the increase in both awareness and attendance over the years, there remains a substantial gap between knowing that screening is available and the actual utilization of the screening services

Additionally, we looked into the study population’s trends on mammography behaviour over the years. From 2002 to 2018, mammography awareness has increased from 70.8 to 91.1%, and the proportion of women who reported ever attending mammography increased from 37.5 to 63.7% (Fig. 1). However, the attendance rate within the recommended screening interval of 2 years is lower (20.8% in 2002 and only increasing to 26% in 2018). Despite the increase in both awareness and attendance over the years, there remains a substantial gap between knowing that screening is available and the actual utilization of the screening services.

### Mammography screening attendance is associated with more favourable breast cancer tumour characteristics at diagnosis

Table 2 shows the associations between mammography behaviour and disease characteristics, adjusted for age at diagnosis, site, ethnicity, and case type (incident/prevalent). Compared to screeners (reference category for all comparisons), non-screeners were significantly more likely to be diagnosed with late-stage cancers (OR_stage II vs stage I (reference)_: 1.72 [1.46–2.02], *p* < 0.001; OR_stage III vs stage I (reference)_: 3.17 [2.52–3.98], *p* < 0.001). This means that the odds of a non-screener developing stage III breast cancer is 3.17 times that of a screener. Non-screeners also showed higher odds of developing high-grade tumours (OR_poorly vs well-differentiated (reference)_: 1.58 [1.26–1.97], *p* < 0.001), positive nodal status (OR_positive vs negative nodal status (reference)_: 1.61 [1.38–1.88], *p* < 0.001), and larger tumour size (OR_>5cm vs ≤2cm (reference)_: 3.22 [2.25–4.61], *p* < 0.001).

**Table 2:** Associations between mammography behaviour and disease characteristics

	Screeners, ***n***=1089		Non-screeners, ***n***=2260			Unaware, ***n***=390		
	***N***		***N***	OR (95% CI)	***P***	***N***	OR (95% CI)	***P***
**Stage**								
I	521	1.00 (reference)	694			90		
II	437		**1011**	**1.72 (1.46–2.02)**	**<0.001**	**194**	**2.72 (2.02–3.65)**	**<0.001**
III	131		**555**	**3.17 (2.52–3.98)**	**<0.001**	**106**	**4.94 (3.45–7.07)**	**<0.001**
**Grade**								
Well-differentiated	190	1.00 (reference)	284			59		
Moderately differentiated	451		**949**	**1.41 (1.13–1.76)**	**0.002**	149	1.15 (0.80–1.65)	0.461
Poorly differentiated	413		**941**	**1.58 (1.26–1.97)**	**<0.001**	**160**	**1.53 (1.06–2.20)**	**0.022**
Missing	35		86			22		
**Nodal status**								
Negative	739	1.00 (reference)	1269			208		
Positive	345		**959**	**1.61 (1.38–1.89)**	**<0.001**	**180**	**1.96 (1.52–2.52)**	**<0.001**
Missing	5		32			2		
**Tumour size**								
≤2cm	643	1.00 (reference)	947			133		
>2–≤5cm	389		**1037**	**1.73 (1.48–2.03)**	**<0.001**	**204**	**2.43 (1.86–3.16)**	**<0.001**
>5cm	40		**205**	**3.22 (2.25–4.61)**	**<0.001**	**44**	**5.06 (3.10–8.25)**	**<0.001**
Missing	17		71			9		
**Oestrogen receptor status**								
Positive	775	1.00 (reference)	1538			277		
Negative	231		514	1.12 (0.93–1.34)	0.225	85	1.10 (0.82–1.48)	0.516
Missing	83		208			28		
**Progesterone receptor status**								
Positive	642	1.00 (reference)	1316			247		
Negative	364		727	1.00 (0.85–1.17)	0.989	116	0.92 (0.70–1.20)	0.54
Missing	83		217			27		
**HER2 status**								
Positive	236	1.00 (reference)	540			89		
Negative	714		**1415**	**0.80 (0.67–0.96)**	**0.016**	**252**	**0.72 (0.53–0.97)**	**0.028**
Missing	139		305			49		
**Subtype**								
Luminal A	445	1.00 (reference)	880			156		
Luminal B [HER2-negative]	159		354	1.15 (0.92–1.44)	0.206	**69**	**1.44 (1.01–2.05)**	**0.041**
Luminal B [HER2-positive]	8		22	1.48 (0.65–3.38)	0.352	6	2.53 (0.81–7.84)	0.109
HER2-overexpressed	86		**219**	**1.39 (1.05–1.83)**	**0.022**	31	1.40 (0.88–2.23)	0.158
Triple negative	107		204	0.96 (0.74–1.25)	0.754	39	1.12 (0.73–1.72)	0.607
Missing	284		581			89		

Odds ratios (OR) and 95% confidence intervals (CI) were estimated using multinomial regression. *P* indicates the *p-*value obtained from the Wald test. The model was adjusted for age at diagnosis, site, ethnicity, and case type (incident/prevalent). Bold indicates statistical significance at *p*-value <.05

Likewise, similar trends were observed among patients who were unaware of mammography. They were associated with increased odds of being diagnosed with later stage cancers (OR_stage II vs stage I (reference)_: 2.72 [2.02–3.65], *p* < 0.001; OR_stage III vs stage I (reference)_: 4.95 [3.45–7.07], *p* < 0.001), high-grade tumours (OR_poorly vs well-differentiated (reference)_: 1.53 [1.06–2.20], *p* = 0.022), positive nodal status (OR_positive vs negative nodal status (reference)_: 1.96 [1.52–2.52], *p* < 0.001), and larger tumour size (OR_>5cm vs ≤2cm (reference)_: 5.06 [3.10–8.25], *p* < 0.001).

In terms of HER2 status, both non-screeners and those who are unaware were less likely to be diagnosed with HER2-negative cancers (non-screeners OR_HER2-negative vs HER2-positive (reference)_: 0.80 [0.67–0.96], *p* = 0.016; unaware OR_HER2-negative vs HER2-positive (reference)_: 0.72 [0.53–0.97], *p* = 0.028). However, there were no significant associations between mammography behaviour and hormone receptor status. Furthermore, when looking at proxy subtypes, non-screeners are at higher odds of developing HER2-overexpressed cancers (OR_HER2-overexpressed vs luminal A (reference)_: 1.39 [1.05–1.83], *p* = 0.022) and patients who are unaware have higher odds of developing luminal B (HER2-negative) cancers (OR_luminal B [HER2-negative] vs luminal A (reference)_: 1.44 [1.01–2.05], *p* = 0.041).

The results did not change appreciably in sensitivity analyses including patients diagnosed with stage 0 or stage IV breast cancer (Additional file 1: Table S2). However, contrary to what we found in the main study population, a subset analysis including only incident breast cancer cases found no significant association between mammography behaviour and HER2 status (non-screeners OR_HER2-negative vs HER2-positive (reference)_: 0.87 [0.68–1.12], *p* = 0.286; unaware OR_HER2-negative vs HER2-positive (reference)_: 0.95 [0.61–1.47], *p* = 0.811) (Additional file 1: Table S3). Furthermore, both non-screeners and those who are unaware were significantly less likely to be diagnosed with PR-negative cancers (non-screeners OR_PR-negative vs PR-positive (reference)_: 0.80 [0.60–0.94], *p* = 0.013; unaware OR_PR-negative vs PR-positive (reference)_: 0.62 [0.41–0.92], *p* = 0.018). Non-screeners among incident cases were also at lower odds of developing triple-negative cancers (OR_triple negative vs luminal A (reference)_: 0.77 [0.49–0.99], *p* = 0.046).

### Mammography screening attendance is associated with more favourable overall cancer survival

**Fig. 2:**
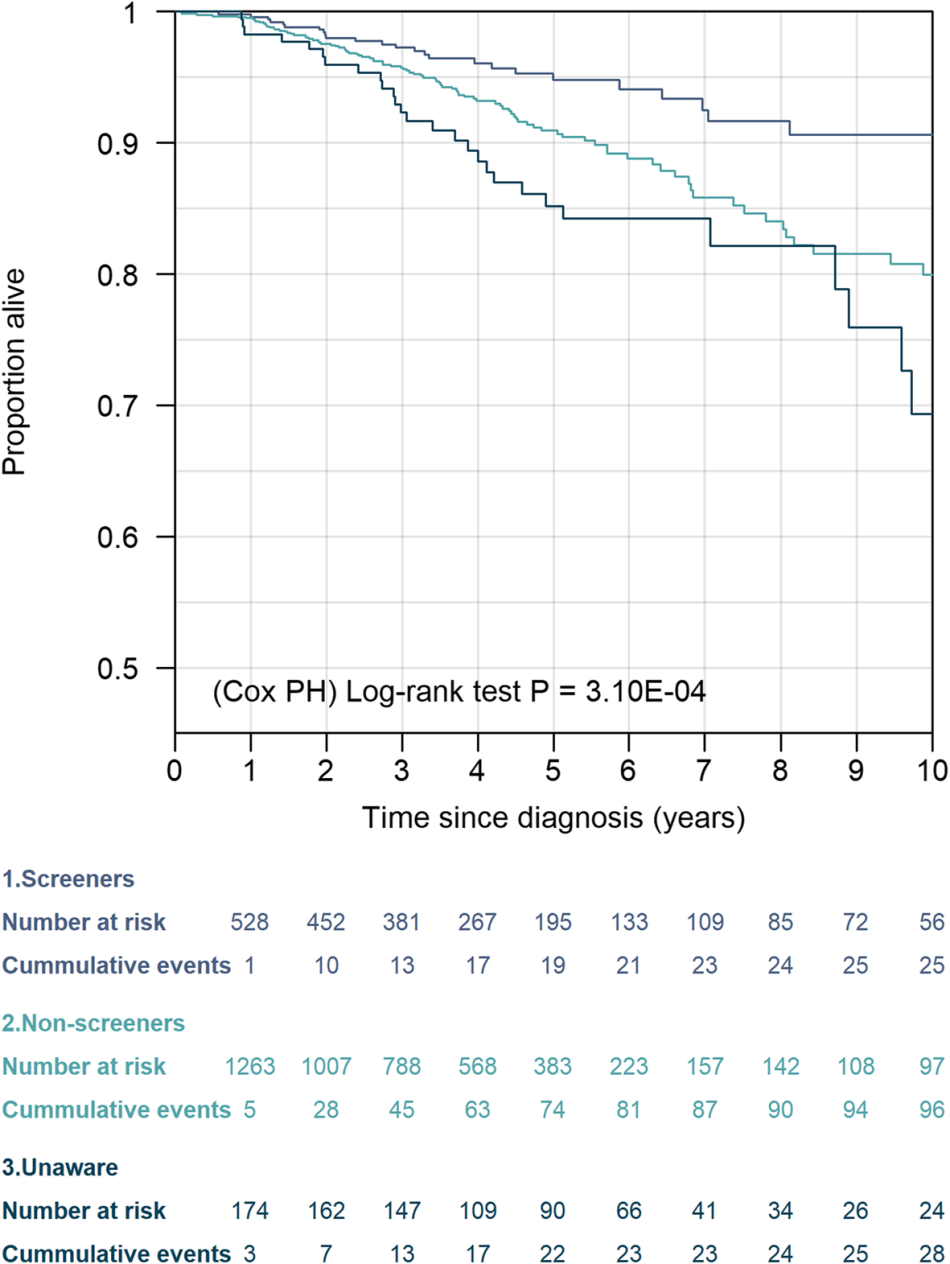
Kaplan-Meier curves for breast cancer patients. Ten-year overall survival is illustrated according to mammography behaviour (screeners, non-screeners, unaware). The *p*-value is a log-rank test

Figure 2 presents the Kaplan-Meier curve for overall survival in 3739 breast cancer patients. A total of 149 deaths occurred within 10 years after diagnosis. In univariate Cox regression, both non-screeners and patients who were unaware were at significantly higher risk of death (HR_non-screeners_ [95% CI]: 1.89 [1.22–2.94], *p* = 0.005; HR_unaware_: 2.90 [1.69–4.98], *p* < 0.001) (Table 3). Adjusted model 1 presents the HR after adjusting for patient characteristics that were significant in the univariate Cox regression models (Additional file 1: Table S4). Even after adjustments, non-screeners were at a significantly higher risk of death compared to screeners (HR_non-screeners_: 1.77 [1.12–2.77], *p* = 0.014). The effect of mammography behaviour on survival was no longer significant after further adjustments with disease and tumour characteristics (adjusted models 2 and 3). In the 5-year survival analyses conducted, similar results were observed (Additional file 1: Table S5 and Fig. S6).

**Table 3:** Association of mammography behaviour with 10-year overall survival

	Univariate		Adjusted model 1		Adjusted model 2		Adjusted model 3	
	HR (95% CI)	***P***	HR (95% CI)	***P***	HR (95% CI)	***P***	HR (95% CI)	***P***
**Mammography behaviour**								
Screeners	1.00 (reference)							
Non-screeners	**1.89 (1.22–2.94)**	**0.005**	**1.77 (1.12–2.77)**	**0.014**	1.48 (0.94–2.31)	0.088	1.44 (0.92–2.25)	0.113
Unaware	**2.90 (1.69–4.98)**	**<0.001**	1.80 (0.99–3.27)	0.054	1.58 (0.89–2.79)	0.118	1.58 (0.89–2.79)	0.118

Hazard ratios (HR) and 95% confidence intervals (CI) were estimated using Cox regression models. Adjusted model 1 was adjusted for all patient characteristics significant in the univariate model (except age at first full-term pregnancy, due to collinearity with parity); adjusted model 2 was adjusted for disease characteristics significant in the univariate models and patient characteristics that remained significant in adjusted model 1; adjusted model 3 was adjusted for treatment characteristics significant in the univariate models and patient and disease characteristics that remained significant in adjusted model 2. Refer to Additional file 1: Table S4 for HR and 95% CI of all variables in the models

Further sensitivity analyses were performed on a subset of the data including incident cases only. Screeners continued to show better 10-year overall survival (original analysis: HR_non-screeners_: 1.89 [1.22–2.94], *p* = 0.005; HR_unaware_: 2.90 [1.69–4.98], *p* < 0.001; incident cases only: HR_non-screeners_: 1.25 [0.68–2.29], *p* = 0.467; HR_unaware_: 2.02 [0.60–4.55], *p* = 0.09). However, the association was no longer significant due to the smaller number of events (Additional file 1: Table S6 and Fig. S7). In contrast, trends observed between mammography behaviour and overall survival among population including those diagnosed with stage 0 or stage IV cancer were more pronounced, where even after adjustments for patient, disease, and treatment characteristics, both non-screeners and those unaware remained at significantly higher risk of death compared to screeners (HR_non-screeners_: 1.57 [1.06–2.33], *p* = 0.026; HR_unaware_: 1.64 [1.00–2.67], *p* = 0.048) (Additional file 1: Table S7 and Fig. S8).

Additional analyses were conducted to assess the differences between non-regular screeners (*n* = 1210, attended mammography but could not recall the year of the last visit/attended mammography but the last visit was more than 2 years prior to diagnosis) and true non-screeners (*n* = 1050, have not attended mammography). Compared to non-regular screeners, true non-screeners were at higher risk of developing late stage (OR_stage III vs stage I (reference)_: 2.11 [1.66–2.67], *p* < 0.001), high-grade tumours (OR_poorly vs well-differentiated (reference)_: 1.52 [1.15–2.01], *p* < 0.001), positive nodal status (OR_positive vs negative nodal status (reference)_: 1.38 [1.16–1.64], *p* < 0.001), and larger tumour size (OR_>5cm vs ≤2cm (reference)_: 2.75 [1.99–3.81], *p* < 0.001), after adjusting for age at diagnosis, site, ethnicity, and case type (incident/ prevalent) (Additional file 1: Table S8). True non-screeners were less likely to be diagnosed with HER2-negative cancers and at higher risk of developing luminal B type cancers (Additional file 1: Table S8). However, there was no difference in overall survival between the two groups (Additional file 1: Fig. S9).

### Screening attendees tend to be younger, received higher education, and have had a family history of breast cancer

Table 4 shows the associations between sociodemographic factors and mammography behaviour, adjusted for age at diagnosis, site, and case type (incident/prevalent). Non-screeners were more likely to be of older age group (OR_≥60 vs 50–59 (reference)_: 1.36 [1.18–1.58], *p* < 0.001), more likely to be Malay (OR_Malay vs Chinese (reference)_: 1.42 [1.11–1.82], *p* = 0.005), have no formal or only primary education (OR_no formal/primary vs secondary (reference)_: 1.76 [1.46–2.11], *p* < 0.001), and residing in 1 to 3 rooms HDB (OR_1–3 rooms HDB vs >3 rooms HDB (reference)_: 1.43 [1.18–1.73], *p* < 0.001). Additionally, they were more likely to be past smokers (OR_smokers vs non-smokers (reference)_: 1.59 [1.01–2.50], *p* = 0.045) and less likely to be physically active (OR_5 vs 2 (reference)_: 0.52 [0.40–0.68], *p* < 0.001). In terms of medical risk factors, they were less likely to have had previous surgery for benign lump (OR_no vs yes (reference)_: 0.50 [0.41–0.62], *p* < 0.001) or gynaecological condition (OR_no vs yes (reference)_: 0.79 [0.68–0.92], *p* = 0.002) and less likely to have family history of breast cancer (OR_no vs yes (reference)_: 0.74 [0.62–0.87], *p* < 0.001). Looking into reproductive risk factors, non-screeners were more likely to be nulliparous (OR_nulliparous vs 25–29 (reference)_: 1.27 [1.03–1.57], *p* = 0.028). Furthermore, they were less likely to have undergone hormone replacement treatment (OR_yes vs no (reference)_: 0.59 [0.47–0.74], *p* < 0.001) compared to screeners.

**Table 4:** Associations between sociodemographic factors and mammography behaviour

	Screeners, ***n***=1089		Non-screeners, ***n***=2260			Unaware, ***n***=390		
	***N***		***N***	OR (95% CI)	***P***	***N***	OR (95% CI)	***P***
**Sociodemographic factors**								
**Age at diagnosis (categorical)**								
50–59	610	1.00 (reference)	1084			106		
≥60	479		**1176**	**1.36 (1.18–1.58)**	**<0.001**	**284**	**3.60 (2.79–4.66)**	**<0.001**
**Ethnicity**								
Chinese	888	1.00 (reference)	1797			315		
Malay	99		**281**	**1.42 (1.11–1.82)**	**0.005**	**51**	**1.89 (1.29–2.77)**	**0.001**
Indian	72		122	0.85 (0.62–1.15)	0.285	21	0.97 (0.58–1.63)	0.906
Others	30		60	0.94 (0.60–1.48)	0.79	**3**	**0.24 (0.07–0.83)**	**0.024**
**Highest qualification attained**								
No formal/primary	241		**837**	**1.76 (1.46–2.11)**	**<0.001**	**280**	**5.04 (3.77–6.75)**	**<0.001**
Secondary	545	1.00 (reference)	1013			93		
Post-secondary (non-tertiary)	84		114	0.75 (0.55–1.01)	0.062	**4**	**0.30 (0.11–0.83)**	**0.021**
Professional diploma	93		**88**	**0.51 (0.37–0.70)**	**<0.001**	**2**	**0.15 (0.04–0.63)**	**0.01**
Tertiary	94		131	0.75 (0.56–1.00)	0.053	**2**	**0.14 (0.03–0.58)**	**0.007**
Missing	32		77			9		
**Housing**								
HDB 1–3 rooms	192		**566**	**1.43 (1.18–1.73)**	**<0.001**	**154**	**2.33 (1.77–3.07)**	**<0.001**
HDB >3 rooms	694	1.00 (reference)	1408			206		
Private	193		**247**	**0.60 (0.48–0.74)**	**<0.001**	**22**	**0.28 (0.17–0.45)**	**<0.001**
Others/missing	10		39			8		
**Marital status**								
Married	828	1.00 (reference)	1595			228		
Never married	126		299	1.25 (0.99–1.57)	0.057	44	1.18 (0.80–1.73)	0.41
Widowed	84		244	1.26 (0.95–1.67)	0.106	**93**	**1.85 (1.28–2.69)**	**0.001**
Separated/divorced	51		122	1.22 (0.86–1.71)	0.261	25	1.57 (0.93–2.65)	0.09
**Lifestyle risk factors**								
**Smoking**								
No	1063	1.00 (reference)	2179			369		
Yes	26		**81**	**1.59 (1.01–2.50)**	**0.045**	**21**	**2.85 (1.53–5.32)**	**<0.001**
**Alcohol**								
No	1055	1.00 (reference)	2194			384		
Yes	34		66	1.00 (0.66–1.53)	0.99	6	0.63 (0.26–1.55)	0.313
**Physical activity between 18 and 30**								
1	70		158	0.90 (0.65–1.25)	0.543	36	0.80 (0.48–1.33)	0.397
2	673	1.00 (reference)	1541			303		
3	78		126	0.75 (0.55–1.02)	0.068	**6**	**0.17 (0.07–0.42)**	**<0.001**
4	146		279	0.89 (0.71–1.11)	0.302	**30**	**0.49 (0.32–0.76)**	**0.001**
5	122		**156**	**0.52 (0.40–0.68)**	**<0.001**	**15**	**0.30 (0.17–0.53)**	**<0.001**
**Medical risk factors**								
**Charlson comorbidity index**								
0	780	1.00 (reference)	1552			217		
1	216		520	1.14 (0.95–1.38)	0.154	**116**	**1.48 (1.11–1.97)**	**0.007**
>1	93		184	0.94 (0.71–1.22)	0.626	**57**	**1.57 (1.07–2.31)**	**0.021**
Missing	0		4			0		
**Previous surgery for benign lump**								
No	896	1.00 (reference)	2038			367		
Yes	193		**221**	**0.50 (0.41–0.62)**	**<0.001**	**21**	**0.28 (0.17–0.45)**	**<0.001**
Missing	0		1			2		
**Previous gynaecological surgery**								
No	632	1.00 (reference)	1414			256		
Yes	456		**844**	**0.79 (0.68–0.92)**	**0.002**	**131**	**0.64 (0.50–0.83)**	**<0.001**
Missing	1		2			3		
**Family history of breast cancer**								
No	760	1.00 (reference)	1687			312		
Yes	294		**482**	**0.74 (0.62–0.87)**	**<0.001**	**61**	**0.54 (0.39–0.74)**	**<0.001**
Missing	35		91			17		
**Family history of ovarian cancer**								
No	1017	1.00 (reference)	2101			359		
Yes	32		62	0.98 (0.63–1.52)	0.918	13	1.37 (0.69–2.72)	0.369
Missing	40		97			18		
**Reproductive risk factors**								
**Age at first full-term pregnancy**								
Nulliparous	200		**462**	**1.27 (1.03–1.57)**	**0.028**	63	1.18 (0.81–1.71)	0.387
<20	45		118	1.31 (0.90–1.90)	0.153	**54**	**3.19 (1.98–5.13)**	**<0.001**
20–24	209		497	1.20 (0.97–1.49)	0.085	**102**	**1.42 (1.01–1.99)**	**0.042**
25–29	349	1.00 (reference)	661			103		
>30	283		510	0.99 (0.81–1.20)	0.908	60	0.86 (0.59–1.24)	0.411
Missing	3		12			8		
**Parity**								
0	200		**462**	**1.37 (1.11–1.68)**	**0.003**	**63**	**1.45 (1.00–2.11)**	**0.049**
1	126		**295**	**1.39 (1.09–1.77)**	**0.008**	38	1.40 (0.90–2.17)	0.136
2	442	1.00 (reference)	762			92		
≥3	321		**741**	**1.25 (1.04–1.50)**	**0.015**	**196**	**2.06 (1.52–2.78)**	**<0.001**
Missing	0		0			1		
**Infertility treatment**								
No	1028	1.00 (reference)	2178			384		
Yes	61		82	0.72 (0.51–1.02)	0.068	6	0.47 (0.20–1.10)	0.083
**Oral contraception**								
No	818	1.00 (reference)	1698			297		
Yes	271		562	0.98 (0.82–1.16)	0.781	93	0.82 (0.62–1.10)	0.185
**Hormone replacement treatment**								
Never	918	1.00 (reference)	2018			373		
Ever	161		**227**	**0.59 (0.47–0.74)**	**<0.001**	**13**	**0.14 (0.08–0.26)**	**<0.001**
Missing	10		15			4		
**Menopausal status at diagnosis**								
Post-menopausal	845	1.00 (reference)	1885			354		
Pre-menopausal	244		375	0.94 (0.76–1.17)	0.608	36	1.27 (0.82–1.95)	0.287

Odds ratios (OR) and 95% confidence intervals (CI) were estimated using multinomial regression. The model was adjusted for age at diagnosis, site, and case type (incident/prevalent). *P* indicates *p*-value obtained from the Wald test. Bold indicates statistical significance at *p*-value <.05

Similarly, those unaware of mammography were more likely to be older (OR _≥60 vs 50–59 (reference)_: 3.60 [2.79–4.66], *p* < 0.001), Malay (OR_Malay vs Chinese (reference)_: 1.89 [1.29–2.77], *p* = 0.001), received no formal or only primary education (OR_no formal/primary vs secondary (reference)_: 5.04 [3.77–6.75], *p* < 0.001), reside in 1 to 3 rooms HDB (OR_1–3 rooms HDB vs > 3 rooms HDB (reference)_: 2.33 [1.77–3.07], *p* < 0.001), and widowed (OR_widowed vs married (reference)_: 1.85 [1.28–2.69], *p* = 0.001). They were associated to be past smokers (OR_smokers vs non-smokers (reference)_: 2.85 [1.53–5.32], *p* < 0.001) and less physically active (OR_5 vs 2 (reference)_: 0.30 [0.17–0.53], *p* < 0.001). In addition, they were more likely to suffer from other comorbidities (OR_CCI>1 vs CCI=0 (reference)_: 1.57 [1.07–2.31], *p* = 0.021), but less likely to have had previous surgery for benign lump (OR_no vs yes (reference)_: 0.28 [0.17–0.45], *p* < 0.001) or gynaecological surgery (OR_no vs yes (reference)_: 0.64 [0.50–0.83], *p* < 0.001) or have had family history of breast cancer (OR_no vs yes (reference)_: 0.54 [0.39–0.74], *p* < 0.001). They were also younger at their first full-term pregnancy (OR_<20 vs 25–19 (reference)_: 3.19 [1.98–5.13], *p* < 0.001) and less likely to have undergone hormone replacement treatment (OR_yes vs no (reference)_: 0.14 [0.08–0.26], *p* < 0.001). The results remained largely unchanged in the sensitivity analysis including incident-only cases (Additional file 1: Table S9).

### Deterrents and motivators of mammography attendance

**Fig. 3:**
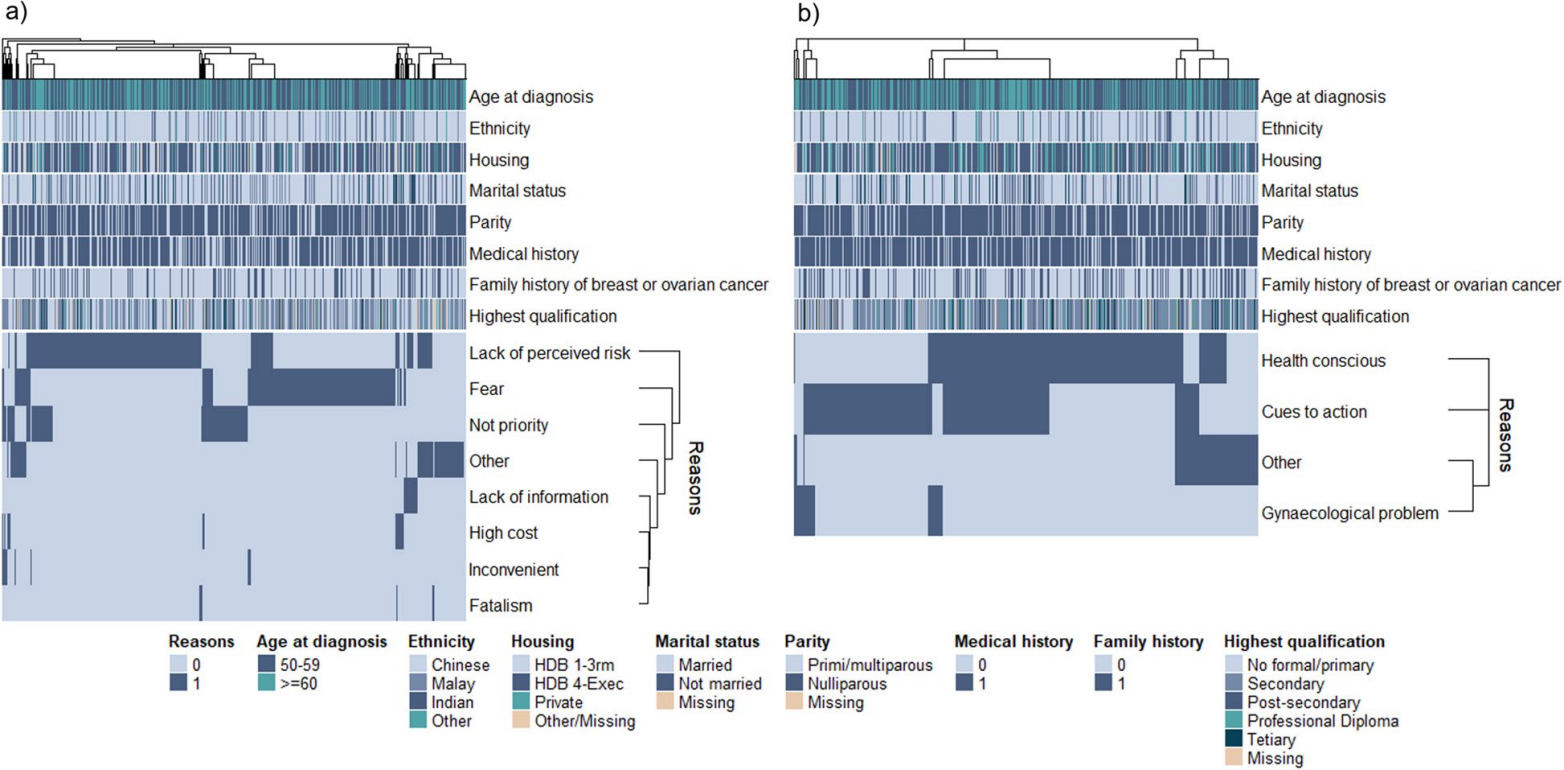
Heatmap showing reasons for mammography **a** non-attendance among non-attendees (*n* = 1050) and **b** attendance among true screeners (*n* = 1089), respectively. The main deterrents were lack of perceived risk and fear, while motivators can be categorized as health consciousness or cues to action. Non-attendees exclude non-regular screeners who indicated attendance but could not recall/last visit was more than 2 years prior to diagnosis

We further looked into the patterns surrounding deterrents and motivators for attending mammography among non-attendees and attendees respectively (Fig. 3). Some major deterrents flagged out were lack of perceived risk by patients, as well as fear (Fig. 3a), which can include fear of screening side effects and fear of diagnosis. However, there were no major patterns identified across the different deterrents.

On the other hand, in terms of motivators, there were distinct groups that can be identified from the heat map (Fig. 3b). The groups were categorized as follows: (1) those who are motivated by both cues and innate health consciousness, (2) those who are motivated solely by appropriate cues to action or (3) solely by innate health consciousness, and (4) others. To better understand ways to improve targeting of appropriate cues to increase screening attendance, we further looked into characteristics of patients who were motivated by cues to action (Table 5). In the univariate model, those who were motivated by cues to action were less likely to be health conscious (OR_health conscious vs not health conscious (reference)_: 0.20 [0.15–0.26], *p* < 0.001), were more likely to be of older age group (OR_≥ 60 vs 50–59 (reference)_: 1.43 [1.12–1.82], *p* = 0.004), have received no formal/only primary education (OR_no formal/primary vs secondary (reference)_: 1.67 [1.21–2.29], *p* = 0.002), reside in 1 to 3 rooms HDB (OR_1–3 rooms HDB vs >3 rooms HDB (reference)_: 1.48 [1.06–2.06], *p* < 0.021), widowed (OR_widowed vs married (reference)_: 1.78 [1.11–2.87], *p* = 0.018) or separated (OR_separated/divorced vs married (reference)_: 2.03 [1.09–3.76], *p* = 0.025), and indicated lower levels of physical activity (OR_5 vs 2 (reference)_: 0.48 [0.32–0.71], *p* < 0.001). In terms of reproductive risk factors, those who were motivated by cues to action were significantly associated with younger age at first full-term pregnancy (OR_<20 vs 25–19 (reference)_: 2.71 [1.30–5.63], *p* = 0.008), have more children (OR_≥3 vs 2 (reference)_: 1.52 [1.13–2.03], *p* = 0.005), and have been on oral contraception (OR_yes vs no (reference)_: 1.54 [1.16–2.04], *p* = 0.003). However, age at diagnosis, highest qualification attained, housing, marital status, physical activity level, parity, use of oral contraception, and menopausal status at diagnosis no longer had a significant effect on whether or not participants were motivated by cues to action after adjustments.

**Table 5:** Characteristics of patients motivated by cues to action

	Not motivated by cues to action, ***n***=483	Motivated by cues to action, ***n***=606	Univariate		Adjusted	
			OR (95% CI)	***P***	OR (95% CI)	***P***
**Site**						
CGH	59	20	1.00 (reference)			
KKH	85	88	**3.05 (1.70–5.50)**	**<0.001**	1.28 (0.64–2.58)	0.487
NCC	80	180	**6.64 (3.75–11.75)**	**<0.001**	**5.10 (2.68–9.73)**	**<0.001**
NTFGH	5	2	1.18 (0.21–6.57)	0.85	0.90 (0.13–6.03)	0.914
NUH	111	53	1.41 (0.77–2.58)	0.266	0.83 (0.41–1.68)	0.6
SGH	48	59	**3.63 (1.92–6.84)**	**<0.001**	1.82 (0.86–3.85)	0.119
TTSH	95	204	**6.34 (3.61–11.12)**	**<0.001**	**4.06 (2.15–7.67)**	**<0.001**
**Health consciousness**						
0	92	330	1.00 (reference)			
1	391	276	**0.20 (0.15–0.26)**	**<0.001**	**0.16 (0.11–0.22)**	**<0.001**
**Sociodemographic factors**						
**Age at diagnosis (categorical)**						
50–59	294	316	1.00 (reference)			
≥60	189	290	**1.43 (1.12–1.82)**	**0.004**	1.28 (0.92–1.77)	0.141
**Ethnicity**						
Chinese	382	506	1.00 (reference)			
Malay	52	47	0.68 (0.45–1.03)	0.072		
Indian	37	35	0.71 (0.44–1.16)	0.17		
Others	12	18	1.13 (0.54–2.38)	0.743		
**Highest qualification attained**						
No formal/primary	78	163	**1.67 (1.21–2.29)**	**0.002**	1.15 (0.78–1.70)	0.471
Secondary	242	303	1.00 (reference)			
Post-secondary (non-tertiary)	34	50	1.17 (0.74–1.87)	0.5	1.27 (0.73–2.20)	0.39
Professional diploma	60	33	**0.44 (0.28–0.69)**	**<0.001**	0.59 (0.35–1.01)	0.054
Tertiary	54	40	**0.59 (0.38–0.92)**	**0.02**	0.86 (0.50–1.47)	0.576
Missing	15	17				
**Housing**						
HDB 1–3 rooms	66	126	**1.48 (1.06–2.06)**	**0.021**	1.09 (0.73–1.64)	0.663
HDB >3 rooms	303	391	1.00 (reference)			
Private	109	84	**0.60 (0.43–0.82)**	**0.002**	0.80 (0.54–1.19)	0.271
Others/missing	5	5				
**Marital status**						
Married	379	449	1.00 (reference)			
Never married	62	64	0.87 (0.60–1.27)	0.472	1.05 (0.54–2.04)	0.887
Widowed	27	57	**1.78 (1.11–2.87)**	**0.018**	1.25 (0.72–2.17)	0.426
Separated/divorced	15	36	**2.03 (1.09–3.76)**	**0.025**	2.07 (0.98–4.35)	0.056
**Lifestyle risk factors**						
**Smoking**						
No	470	593	1.00 (reference)			
Yes	13	13	0.79 (0.36–1.73)	0.559		
**Alcohol**						
No	468	587	1.00 (reference)			
Yes	15	19	1.01 (0.51–2.01)	0.978		
**Physical activity between 18 and 30**						
1	32	38	0.82 (0.50–1.35)	0.433	1.15 (0.61–2.18)	0.664
2	275	398	1.00 (reference)			
3	40	38	0.66 (0.41–1.05)	0.079	0.58 (0.33–1.03)	0.062
4	64	82	0.89 (0.62–1.27)	0.509	0.99 (0.65–1.52)	0.972
5	72	50	**0.48 (0.32–0.71)**	**<0.001**	0.70 (0.44–1.12)	0.135
**Medical risk factors**						
**Charlson comorbidity index**						
0	354	426	1.00 (reference)			
1	96	120	1.04 (0.77–1.41)	0.806		
>1	33	60	1.51 (0.97–2.36)	0.071		
**Previous surgery for benign lump**						
No	394	502	1.00 (reference)			
Yes	89	104	0.92 (0.67–1.25)	0.587		
**Previous gynaecological surgery**						
No	291	341	1.00 (reference)			
Yes	192	264	1.17 (0.92–1.50)	0.198		
Missing	0	1				
**Family history of breast cancer**						
No	336	424	1.00 (reference)			
Yes	135	159	0.93 (0.71–1.22)	0.617		
Missing	12	23				
**Family history of ovarian cancer**						
No	458	559	1.00 (reference)			
Yes	12	20	1.37 (0.66–2.82)	0.401		
Missing	13	27				
**Reproductive risk factors**						
**Age at first full-term pregnancy**						
Nulliparous	99	101	0.79 (0.56–1.12)	0.179	0.89 (0.66–1.21)	0.467
<20	10	35	**2.70 (1.30–5.63)**	**0.008**	1.82 (0.77–4.30)	0.174
20–24	93	116	0.96 (0.68–1.36)	0.828	**0.65 (0.43–1.00)**	**0.048**
25–29	152	197	1.00 (reference)			
>30	127	156	0.95 (0.69–1.30)	0.739	1.00 (0.68–1.47)	0.986
Missing	2	1				
**Parity**						
0	99	101	0.92 (0.66–1.29)	0.64	0.89 (0.66–1.21)	0.467
1	54	72	1.21 (0.81–1.80)	0.356	1.04 (0.65–1.68)	0.863
2	210	232	1.00 (reference)			
≥3	120	201	**1.52 (1.13–2.03)**	**0.005**	1.04 (0.72–1.49)	0.847
**Infertility treatment**						
No	455	573	1.00 (reference)			
Yes	28	33	0.94 (0.56–1.57)	0.802		
**Oral contraception**						
No	384	434	1.00 (reference)			
Yes	99	172	**1.54 (1.16–2.04)**	**0.003**	1.25 (0.88–1.77)	0.218
**Hormone replacement treatment**						
Never	412	506	1.00 (reference)			
Ever	67	94	1.14 (0.81–1.60)	0.442		
Missing	4	6				
**Menopausal status at diagnosis**						
Post-menopausal	355	490	1.00 (reference)			
Pre-menopausal	128	116	**0.66 (0.49–0.87)**	**0.004**	0.76 (0.52–1.12)	0.166

Odds ratios (OR) and 95% confidence intervals (CI) were estimated using multinomial regression. *P* indicates *p*-value obtained from the Wald test. The adjusted model was adjusted for all factors significant in the univariate model. Bold indicates statistical significance at *p*-value <.05

*CGH* Changi General Hospital, *KKH* KK Women’s and Children’s Hospital, *NCC* National Cancer Centre, *NTFGH* Ng Teng Fong General Hospital, *NUH* National University Hospital, *SGH* Singapore General Hospital, *TTSH* Tan Tock Seng Hospital

## Discussion

In this large study of 3739 breast cancer patients recruited in the Singapore Breast Cancer Cohort, mammography screening attendance was associated with more favourable breast cancer tumour characteristics at diagnosis. Significantly worse overall survival was observed for both non-screeners and patients who had not heard of mammography screening before their cancer diagnosis. For the former, the associations remained significant even after adjusting for patient characteristics, but the effect sizes were attenuated and associations were no longer significant after tumour and characteristics were included in the model. Notable deterrents for attending mammography screening were identified to be lack of perceived risk and fear of side effects and cancer diagnosis. Among the motivating factors for mammography screening, four main clusters of “screening personalities” emerged: (i) those who are motivated by both extrinsic cues and innate health consciousness, (ii) those who are motivated solely by appropriate cues to action, (iii) those who are motivated by innate health consciousness, and (iv) others. When breast cancers are presented later for treatment, they are more likely to be associated with advanced stage, poor prognosis, and higher treatment cost [19, 20]. Our observation that tumours detected among recent screeners (as a proxy for screen-detected cancers) have more favourable characteristics and confer better survival than tumours detected among non-recent screeners and those unaware of screening suggests that early detection by mammography surveillance does show the benefit of picking up less advanced and less deadly cancers.

In our study, we observed that compared to screeners, non-screeners and those unaware were significantly less likely to develop HER2-negative cancers and were significantly more likely to develop HER2-overexpressed breast cancers. No significant association was found between mammography behaviour and oestrogen or progesterone receptor status. Our finding that non-screeners are more likely to be HER2-overexpressed is consistent with existing literature. An Irish population-based study (*n* = 7161) found that compared to women with screen-detected cancer, non-participants of screening programme were more likely to develop HER2-overexpressing or triple-negative subtype, accompanied with poorer prognosis [21]. In our population, non-screeners and those unaware have significantly higher parity and were more likely to have a history of benign lump compared to screeners, both of which are factors linked to an increased risk of HER2-overexpressed subtype [22, 23].

Our study did not find a significant association between ER status and screening behaviour. In contrast, Niraula et al. studied 1687 breast cancer diagnoses in 69,025 women and found that breast cancers that were not screen-detected (interval breast cancers, non-programme-detected cancers, and noncompliant cancers) were significantly more likely to be ER-negative [24]. The discrepancy in our findings and what is reported in the literature may be due to other considerations, factors such as mammographic density and body mass index. These factors play important roles in determining the molecular subtypes and hormone receptor statuses of breast cancer [22]. However, we were unable to properly account for the effects of these factors due to the lack of information. Additionally, our definitions of subgroups are different from those in the various studies mentioned, which can contribute to the differences in results. Importantly, we did not have information as to whether or not the screeners had screen-detected cancer or diagnostic-detected cancer, which is one of the main criteria in differentiating the subgroups [24].

Screeners have been consistently shown to be associated with a survival benefit [5, 6]. In agreement, screeners in our study exhibited improved overall survival compared to their counterparts. It should be noted that the better overall survival experienced by screeners could be attributed to a number of reasons, such as better prognosis, sociodemographic and lifestyle factors, and treatment adherence. A study done by He et al. showed that non-participants of mammography were more likely to discontinue adjuvant hormone therapy and subsequently experience worse prognosis compared to screening participants [25]. In our study, we observed that even after adjusting for disease and treatment characteristics, screeners continued to show better overall survival, although the association was no longer significant. This implies that mammography behaviour as well as sociodemographic factors may play a bigger role in determining survival compared to disease and treatment characteristics in our study population. Due to the type of treatment information collected, we were unable to explore the relationship between mammography behaviour and treatment adherence in a more in-depth manner.

Despite widespread knowledge about mammography (94.4%), the proportion of women of the appropriate age group (50 to 69 years old) who get screened for breast cancer (38.7%) remains below the ideal participation rate to see a significant reduction in breast cancer mortality at the population level [7, 26]. Several studies have examined the reasons that contribute to the low response to mammography screening in Singapore using qualitative and quantitative approaches. Data from a prospective survey by Straughan and Seow highlighted fatalistic attitudes, perceived barriers, and perceived efficacy of early detection as significant predictors of free mammography screening uptake in the National Breast Screening Project [27]. The authors also noted the importance of social support from the family helped to improve screening behaviour. In a separate study by Straughan and Seow using a focus group approach to uncover barriers and motivators of mammography screening among Chinese women in Singapore, similar conclusions were drawn. Fatalistic attitudes, misinformation regarding the screening modality, and perceived costs (not limited to financial considerations, including burdens of time, effort, and psychological stress) were found to impede screening behaviour [28]. In contrast, confidence in medicine and the influence of informal social support networks to view mammography screening positively facilitated screening behaviour [28].

In addition, Straughan et al. conducted a survey-based study which was administered in-person to 300 attenders and 260 non-attenders to reveal factors contributing to the acceptance of mammographic screening among women in Singapore [29]. It was observed that being Chinese, employment outside the home, history of attending a screening for other conditions, perceived risk of developing cancer, and encouragement from family members are predictors of mammography screening attendance [29]. In yet another study using questionnaire data administered to 208 cancer-free Asian women in Singapore, Teo et al. reported lack of time and cost to be the leading deterrents for attending mammography screening [30]. The authors observed that “being Chinese, having higher education, mammography knowledge, positive motivator scores, and receiving reminders were predictors to regular mammography” [30]. Seetoh et al. reiterated the same factors (i.e. cost of screening, ethnicity, prior screening history, and attitudes towards mammography screening) to be predictors of mammography screening attendance in the results of a quasi-randomized pragmatic trial [31]. In other studies, misconceptions related to screening (pain and discomfort), cost, efficacy, and fatalistic beliefs were found to be recurring themes [32, 33]. The overlapping themes and predictors identified by the various studies and our results suggest that barriers to mammography screening have remained similar and have persisted over the years despite targeted efforts.

In a meta-analysis by Yabroff et al. which included 63 interventions in 43 studies based in the USA, it was reported that behavioural strategies (i.e. strategies that alter cues or stimuli associated with screening behaviour, such as reminders to screen via telephone or mail by healthcare professionals) increased screening by 13.2% compared with non-intervention [34]. In addition, the authors examined cognitive interventions (i.e. provide new information and education, increase existing knowledge, and clarify misperceptions) and sociological interventions (i.e. social norms or peers). The results showed that cognitive interventions using generic education strategies had little impact on screening, but those that used theory-based education (e.g. health belief model), especially when delivered interactively, increased rates by 23.6% when compared to the no intervention group. Sociological interventions were also found to increase screening rates by ~12.6%. Improvements in mammography utilization using these interventions will largely depend on the subgroups in different study populations. The distinct behavioural patterns (22.9% motivated by both innate health consciousness and extrinsic cues, 27.1% motivated solely by innate health consciousness, 24.3% motivated solely by extrinsic cues, and 25.6% motivated by a combination of other factors) among screeners who have had recent mammography support the use of a mixture of different approaches to improve rates of ongoing screening. Nonetheless, the success of targeted interventions among those who are either non-regular screeners or those who are unaware of mammography screening remains to be seen.

The main merit of this study is the large study cohort from multiple hospital sites in Singapore that see a majority of the breast cancer patients in the country. The availability of detailed sociodemographic, screening behaviour, clinical and survival data for the same study population is an added advantage that helps in giving a comprehensive overview of mammography screening behaviour and the associations with disease characteristics and survival. The organized population-based mammography screening programme in Singapore, which is heavily subsidized, reduces the likelihood of selection bias resulting from the accessibility and cost of screening. Additionally, the clinical data of breast cancer characteristics and outcomes were well kept and retrieved from well-maintained electronic databases, accounting for little missing data. Loss to follow-up due to emigration is expected to be minimal for the duration of the study.

Although the group of breast cancer patients classified as screeners had their most recent mammogram in the past 2 years, information was unavailable as to whether it was the first screen, and whether or not the tumour was screen-detected. While mammography screening behaviour can be correlated to tumour characteristics and survival by sampling breast cancer patients, self-selection bias may occur. Women who are at higher risk of developing breast cancer, such as those with a family history of the disease (breast cancer patients have a higher load of this familial risk), may actively choose to attend mammography screening [35]. However, we did not observe an excess of recent screeners in our study population compared to the national average. Additionally, sociodemographic information collected through the questionnaire was not optimized for this study. As a result, there might be over- or under-estimations of the role of various factors (education, income, and social support) on mammography behaviour. To establish a more direct relationship between these factors and mammography or health-seeking behaviour, a validated questionnaire should be used instead. Due to the nature of data collected, we did not have information on breast cancer-specific mortality and could only rely on data on all-cause mortality. However, in Singapore, around 76% of breast cancer patients die of breast cancer, making breast cancer the most common cause of death amongst breast cancer patients [36]. Hence, all-cause mortality remains as a good estimation for this study population. Moreover, there could be residual confounding and effect modification that could have been missed with our study design. As with all the other epidemiological studies, a causal relationship cannot be conclusively drawn because of various potential confounders. For example, the possibility that the lower cancer stage associated with better mammography behaviour (i.e. screeners) may be attributed to other factors, such as their lifestyle (diet, physical activity levels), cannot be excluded. To establish a causal effect of mammography behaviour on breast cancer, large randomized trials should be planned.

Furthermore, being a case-only retrospective study, recall bias and selection bias cannot be eliminated. We were also unable to fully evaluate the effectiveness of attending mammography screening. However, we were able to derive other plausible benefits of screening, such as that of being diagnosed at earlier stages of disease. As this study involves the evaluation of screening effectiveness, common screening programme-related biases such as lead-time, length-time, and immortal time bias must be considered when interpreting the results [37]. To address these biases, several analytical approaches were taken. Firstly, we explored the association between mammography behaviour and survival amongst incident cases only. The slight differences in the results observed from the different subsets suggest that survivor bias cannot be eliminated, and should be considered during the interpretation of the results. Secondly, disease characteristics, such as stage at diagnosis, were adjusted for in the survival analyses. However, the results from these additional analyses were not appreciably different.

## Conclusions

In summary, our results show that mammography screening is associated with both better breast cancer tumour features and survival and that the survival benefit is largely a result of the better tumour characteristics. However, the nationwide screening mammography service is currently underutilized and various studies, including ours, looking into mammography screening behaviour have highlighted largely similar concerns and barriers to entry. A shift in focus to how to tailor interventions to meet individual healthcare needs is needed to increase the number of breast cancers detected early and achieve positive health outcomes.

## Supplementary Information

**Additional file 1: **Supplementary tables and figures. **Figure S1.** Categorization of motivators and deterrents to mammography. **Figure S2.** Physical activity scores. **Figure S3.** Details on deriving menopausal status at diagnosis. **Figure S4.** Categorization of housing and highest qualification achieved. **Figure S5.** Flow chart of study population, which is comprised of breast cancer patients in the Singapore Breast Cancer Cohort (SGBCC), recruited between 2011 and 2018. **Table S1.** Treatment characteristics of study population. **Table S2.** Associations between mammography behaviour and disease characteristics adjusted for age at diagnosis, site, ethnicity, and case type (incident/prevalent), for population including stages 0 to IV (*n*=4566). **Table S3.** Associations between mammography behaviour and disease characteristics adjusted for age at diagnosis, site, ethnicity, for incident cases (*n*=2122). **Table S4.** Association of patient, tumor and treatment characteristics with ten-year overall survival (*n*=3739). **Table S5.** Association of mammography behaviour with five-year overall survival (*n*=3191). **Figure S6.** Five-year overall survival is illustrated according to mammography behaviour (screeners, non-screeners, unaware). **Table S6.** Association of mammography behaviour with ten-year overall survival, for incident cases (*n*=2122). **Figure S7.** Ten-year overall survival is illustrated according to mammography behaviour (screeners, non-screeners, unaware) for incident cases (*n*=2122). **Table S7.** Association of mammography behaviour with ten-year overall survival, for population including stages 0 to IV (*n*=4566). **Figure S8.** Ten-year overall survival is illustrated according to mammography behaviour (screeners, non-screeners, unaware), for population including stages 0 to IV (*n*=4566). **Table S8.** Comparison between mammography behaviour and disease characteristics adjusted for age at diagnosis, site, ethnicity among non-regular screeners (*n*=1210), and true non-screeners (*n*=1050). **Figure S9.** Ten-year overall survival is illustrated according to mammography behaviour (non-regular screeners, *n*=1210, and true non-screeners, *n*=1050). **Table S9.** Associations between sociodemographic factors and mammography behaviour,adjusted for age at diagnosis and site, for incident cases (*n*=2122).

## Data Availability

Due to ethical reasons and institutional guidelines, the data presented in the study cannot be shared publicly. For ethical issues, please contact the National Healthcare Group Domain Specific Review Board (Email: OHRPP@nhg.com.sg) and the SingHealth Centralised Institutional Review Board (Email: irb@singhealth.com.sg). Data are available to interested researchers with some access restrictions applied upon request. All requests can be directed to the Singapore Breast Cancer Cohort (SGBCC) scientific steering committee. Interested researchers may contact the principal investigator, Mikael Hartman, at mikael_hartman@nuhs.edu.sg for more details. The list of available data can be found in https://blog.nus.edu.sg/sgbcc/for-researchers/

## Abbreviations

BC: Breast cancer
BMI: Body mass index, kg/m^2^
CCI: Charlson comorbidity index
CI: Confidence interval
ER: Oestrogen receptor
HER2: Human epidermal growth factor receptor 2
HR: Hazard ratio
MAM: Mammography screening
OR: Odds ratio
PR: Progesterone receptor
SD: Standard deviation
